# Analysis of the impact of the absence of RAD51 strand exchange activity in Arabidopsis meiosis

**DOI:** 10.1371/journal.pone.0183006

**Published:** 2017-08-10

**Authors:** Gunjita Singh, Olivier Da Ines, Maria Eugenia Gallego, Charles I. White

**Affiliations:** Génétique, Reproduction et Dévelopement, UMR CNRS 6293 - INSERM U1103 - Université Cleront Auvergne Campus Universitaire des Cézeaux, Aubiere, France; Tulane University Health Sciences Center, UNITED STATES

## Abstract

The ploidy of eukaryote gametes must be halved to avoid doubling of numbers of chromosomes with each generation and this is carried out by meiosis, a specialized cell division in which a single chromosomal replication phase is followed by two successive nuclear divisions. With some exceptions, programmed recombination ensures the proper pairing and distribution of homologous pairs of chromosomes in meiosis and recombination defects thus lead to sterility. Two highly related recombinases are required to catalyse the key strand-invasion step of meiotic recombination and it is the meiosis-specific DMC1 which is generally believed to catalyse the essential non-sister chromatid crossing-over, with RAD51 catalysing sister-chromatid and non-cross-over events. Recent work in yeast and plants has however shown that in the absence of RAD51 strand-exchange activity, DMC1 is able to repair all meiotic DNA breaks and surprisingly, that this does not appear to affect numbers of meiotic cross-overs. In this work we confirm and extend this conclusion. Given that more than 95% of meiotic homologous recombination in Arabidopsis does not result in inter-homologue crossovers, Arabidopsis is a particularly sensitive model for testing the relative importance of the two proteins—even minor effects on the non-crossover event population should produce detectable effects on crossing-over. Although the presence of RAD51 protein provides essential support for the action of DMC1, our results show no significant effect of the absence of RAD51 strand-exchange activity on meiotic crossing-over rates or patterns in different chromosomal regions or across the whole genome of Arabidopsis, strongly supporting the argument that DMC1 catalyses repair of all meiotic DNA breaks, not only non-sister cross-overs.

## Introduction

The process of eukaryotic sexual reproduction is based on the production of gametes of halved ploidy, the fusion of two of which regenerates the original ploidy in the subsequent generation [1, 2]. This halving of chromosome number is carried out by meiosis, a specialised cell division in which two successive divisions follow a single round of DNA replication. A single meiotic cell thus produces four nuclei of halved ploidy, in contrast to mitosis, in which DNA replication is followed by a single division, resulting in two daughter nuclei of the same ploidy as the mother cell.

The specialised meiotic cell division thus solves the problem of maintaining ploidy stable across sexual generations, but this comes with a cost. In mitosis, balanced segregation of chromatids, is ensured by sister chromatid cohesion established in the preceding S-phase, but this can only work once and is thus not sufficient in meiosis, in which two successive nuclear divisions follow a single S-phase. In most studied eukaryotes, the problem of proper meiotic chromosomal segregation is ensured by chiasmata, physical links between homologous chromosomes produced by non-sister-chromatid cross-over recombination (CO) in the first meiotic division. Recombination during the first meiotic prophase thus ensures that homologous chromosomes accurately segregate from each other and in doing so, shuffles the genetic information to generate the genetic variation driving evolution.

The work of many authors has contributed to understanding the molecular processes underlying the repair of programmed meiotic DNA double-strand breaks (DSB) and the relationships between CO and non-CO meiotic recombination outcomes. Readers are directed to [3–5] for recent reviews of this subject. Briefly, the process of meiotic recombination is initiated by the programmed induction of DSB throughout the genome by the SPO11 protein complex, followed by resection of the broken DNA ends to generate 3’ single-stranded DNA (ssDNA) overhangs on both sides of the DSB. Binding of the RAD51 and DMC1 proteins to these overhangs generates nucleoprotein filaments, which search for and invade an homologous template DNA duplex. Copying of the template DNA molecule and resolution of the joint recombination intermediates repairs the break. A subset of these repair events result in physical exchanges or CO between the interacting DNA molecules and if these are non-sister chromatids, in chiasmata linking the homologous chromosomes and genetic CO. Strikingly, numbers of meiotic DSB commonly exceed numbers of chiasmata, with DSB:CO ratios of 25–30 in Arabidopsis, 15 in mouse, 4.4 in Drosophila and 1.8 in budding yeast (reviewed by [6]).

The highly conserved RAD51 protein family consists of 7 members in plants and animals: RAD51, DMC1 and the five RAD51 paralogs: RAD51B, RAD51C, RAD51D, XRCC2 and XRCC3. RAD51 and DMC1 catalyse the key recognition and invasion of a homologous DNA template molecule, with the 5 RAD51 paralogs playing essential roles in supporting this activity [7–11]. Originally identified in yeast [12–16], RAD51 and DMC1 are believed to derive from Archaeal *RadA* through a duplication early in eukaryotic evolution [17–19]. The two proteins are weak DNA-dependent ATPases with similar biochemical properties. Binding to ssDNA and dsDNA to form nucleoprotein filaments, which catalyse the search for, and invasion of a homologous DNA template molecule [3, 20–26]. The activities of the two proteins are not however identical, as illustrated by the observation of greater resistance to dissociation of D-loops formed by human DMC1 compared to RAD51 [27] and the differing substrate requirements for the formation of four-strand joint molecules—suggesting opposite polarities of polymerization of RAD51 (3'-5') and DMC1 (5'-3') on ssDNA (Murayama et al. 2011) discussed by [3].

RAD51 plays key roles in both meiosis and mitosis, while DMC1 is meiosis-specific [12, 16]. In meiosis, RAD51 is generally believed to play roles chiefly in inter-sister and non-CO recombination, with DMC1 being important for recombination between non-sister chromatids of homologs, although it can catalyse inter-sister/non-CO recombination in the absence of RAD51 activity [28–31]. Budding yeast *dmc1* mutants arrest in meiotic prophase, accumulate meiotic DSB and have strong defects in accumulation of joint molecule (JM) recombination intermediates [12, 28, 32]. Return to growth experiments do permit recovery of JM intermediates in the yeast *dmc1* mutant, but these are only between sister chromatids [28]. Meiotic prophase arrest is not observed in the yeast *rad51* mutant, which does however show delayed appearance of JM intermediates with a strong bias towards inter-sister versus inter-homologue JM [28] and produces viable spores. The severity of the *dmc1* and *rad51* meiotic phenotypes in yeast is however strain-dependent [33–35].

In mouse, *dmc1* meiosis shows zygotene arrest without synapsis [36, 37], while absence of RAD51 is embryonic lethal [38, 39]. A recent study has succeeded in testing the effects of RAD51 knockdown in mouse meiosis through injection of siRNA into seminiferous tubules and shows leptotene arrest and loss of zygotene nuclei through p53-dependent apoptosis [40]. A few cells escape this apoptosis and these show increased sex-chromosome asynapsis and reduced CO, further supporting the conclusion that RAD51 is needed for DMC1 to function in mouse [40].

Maize has two redundant *RAD51* genes, *RAD51A1* and *RAD51A2* [41]. *rad51a rad51b* mutant plants are viable with no visible developmental defects, but are male sterile with reduced numbers of chiasmata and evidence of non-homologue synapsis in male meiosis. Residual female fertility however permitted apparently normal CO rates in surviving meiocytes [42]. The *japonica* cultivar of rice has two RAD51 proteins (RAD51A1 and RAD51A2) with *in vitro* data suggesting RAD51A2 has the major role in homologous pairing, while *indica* rice plants have only one RAD51 [43, 44]. Rice also has two redundant DMC1 proteins (DMC1A and DMC1B) and rice DMC1 is required for normal meiotic recombination, proper CO formation and synapsis [45–49]. It is however the model plant *Arabidopsis thaliana*, which provides the most clear illustration of the different meiotic phenotypes of *dmc1* and *rad51* mutants. Arabidopsis plants lacking either protein are viable and complete meiosis, but achiasmate meiosis leads to random segregation of intact (fully repaired) chromosomes and residual fertility in the *dmc1* mutant. In striking contrast, the lack of DSB repair leads to meiotic Prophase I chromosome fragmentation in the fully sterile *rad51* mutant [50, 51].

A considerable body of data thus points to a specific role for DMC1 in meiotic inter-homologue CO recombination, but the complexity of the mutant phenotypes has complicated clarification of the specific roles of RAD51 and DMC1 in this process. Recent data from yeast and Arabidopsis have however provided a major advance in sorting out this puzzle. Inactivation of the secondary DNA binding site of RAD51 in *rad51-II3A* mutant yeast blocks its ability to catalyse recombination but does not affect fertility [30]. This is also seen upon expression of the dominant-negative RAD51-GFP fusion protein in Arabidopsis [31], which also lacks secondary DNA binding and strand-invasion activity [52]. In contrast to the effect of absence of RAD51, these mutant RAD51 proteins are unable to catalyse invasion of the template DNA duplex and are defective in mitotic DSB repair, but remain able to support the activity of DMC1 in meiosis [30, 31, 52]. These studies unequivocally show that DMC1 is capable of catalysing the repair of all meiotic DSB in the absence of RAD51 strand-exchange activity. Given the excess of meiotic DSB over CO and the general belief that DMC1 is specifically responsable for meiotic inter-homologue CO recombination, both yeast and plant studies tested for effects on meiotic CO rates. No effect on CO was found in the defined genetic intervals used for these tests, suggesting that DMC1 is the only active strand-invasion enzyme in meiosis and that only the presence of RAD51 is essential, not its strand-exchange activity.

All meiotic recombination is catalysed by DMC1 in the (fully fertile) *rad51* + *RAD51-GFP* Arabidopsis plants, and they thus provide an opportunity for better understanding of the specificities of the roles of DMC1 and RAD51 in inter-homologue meiotic CO and pairing. We present here an analysis of the effects of the absence of RAD51 strand-exchange activity on meiotic CO patterns in different chromosomal regions and across the whole Arabidopsis genome. We find no significant effect of the absence of RAD51 strand-exchange activity on meiosis in Arabidopsis—arguing that DMC1 is the unique active meiotic strand-exchange protein in WT plants.

## Results

### Recombination rates

FTL marker lines [53, 54] were used to test for effects of the absence of RAD51 strand exchange activity on meiotic CO rates in pericentromeric regions. The pollen-expressed, red and yellow fluorescent protein markers in these lines provide a rapid and precise means of measuring genetic map-distance in defined genetic intervals in Arabidopsis. We used the FTL lines I1b carrying linked insertions on the arm of chromosome 1 (I1b: FTL567and FTL1262, and; FTL567: FTL1262 = 8.16 cM), and CEN3, with two insertions spanning the centromere of chromosome 3 (CEN3: FTL3332: FTL2536 = 11.04 cM) [54] (S1 Fig). The I1b and CEN3 lines were crossed with Col-0 WT and *rad51/rad51 RAD51-GFP/RAD51-GFP* homozygotes to generate F1 lines in which both DMC1 and RAD51 (WT), or only DMC1 (*rad51/RAD51 RAD51-GFP*) strand exchange activities are present during meiosis. F2 plants were derived by selfing the F1 and genotyped to identify the *RAD51/RAD51* and *rad51*/*rad51 RAD51-GFP/RAD51-GFP* F2 mapping lines.

Pollen from the WT and *rad51 RAD51-GFP* mapping lines were scored for the fluorescent markers and to guard against biases in scoring, the 1:1 ratio of presence/absence of the markers individual markers was verified with a Chi-squared test in each data set (Tables 1 and 2).

**Table 1:** Meiotic recombination in the CEN3 interval.

Plant#	R	Y	R+Y	neither	total	r	Chi2 R:not R	Chi2 Y:not Y
WT#1	74	68	514	550	1206	0.118	0.750	1.460
WT#2	52	40	360	335	787	0.117	1.740	0.210
WT#3	72	68	544	520	1204	0.116	0.651	0.332
WT#4	103	107	798	772	1780	0.118	0.272	0.506
WT#5	75	78	594	580	1327	0.115	0.091	0.218
RAD51-GFP#1	45	40	312	320	717	0.119	0.010	0.240
RAD51-GFP#2	46	30	285	290	651	0.117	0.190	0.680
RAD51-GFP#3	66	70	542	512	1190	0.114	0.568	0.971
RAD51-GFP#4	75	80	614	590	1359	0.114	0.266	0.619
RAD51-GFP#5	78	65	534	548	1225	0.117	0.001	0.595

**Table 2:** Meiotic recombination in the I1b interval.

Plant#	R	Y	R+Y	neither	total	r	Chi2 R:not R	Chi2 Y:not Y
WT#1	31	20	276	275	602	0.085	0.239	0.166
WT#2	45	36	436	438	955	0.085	0.051	0.013
WT#3	54	50	628	630	1362	0.076	0.003	0.026
WT#4	53	62	645	627	1387	0.083	0.058	0.526
WT#5	35	42	386	402	865	0.089	0.612	0.094
WT#6	54	43	486	507	1090	0.089	0.092	0.939
RAD51-GFP#1	32	23	273	264	592	0.093	0.547	0.000
RAD51-GFP#2	49	51	447	451	998	0.100	0.036	0.004
RAD51-GFP#3	45	24	437	414	920	0.075	2.104	0.004
RAD51-GFP#4	62	41	556	552	1211	0.085	0.516	0.239
RAD51-GFP#5	90	63	842	830	1825	0.084	0.833	0.123
RAD51-GFP#6	60	55	637	625	1377	0.084	0.210	0.036

Numbers of Red (R), Yellow (Y) and Red+Yellow (R+Y) fluorescent and non-fluorescent (neither) pollen from flowers of wild-type and *rad51 RAD51-GFP* plants used to calculate genetic map distances (r cM) in the CEN3 (a) and I1b (b) marked intervals in WT and *RAD51-GFP* plants.

**Fig 1:**
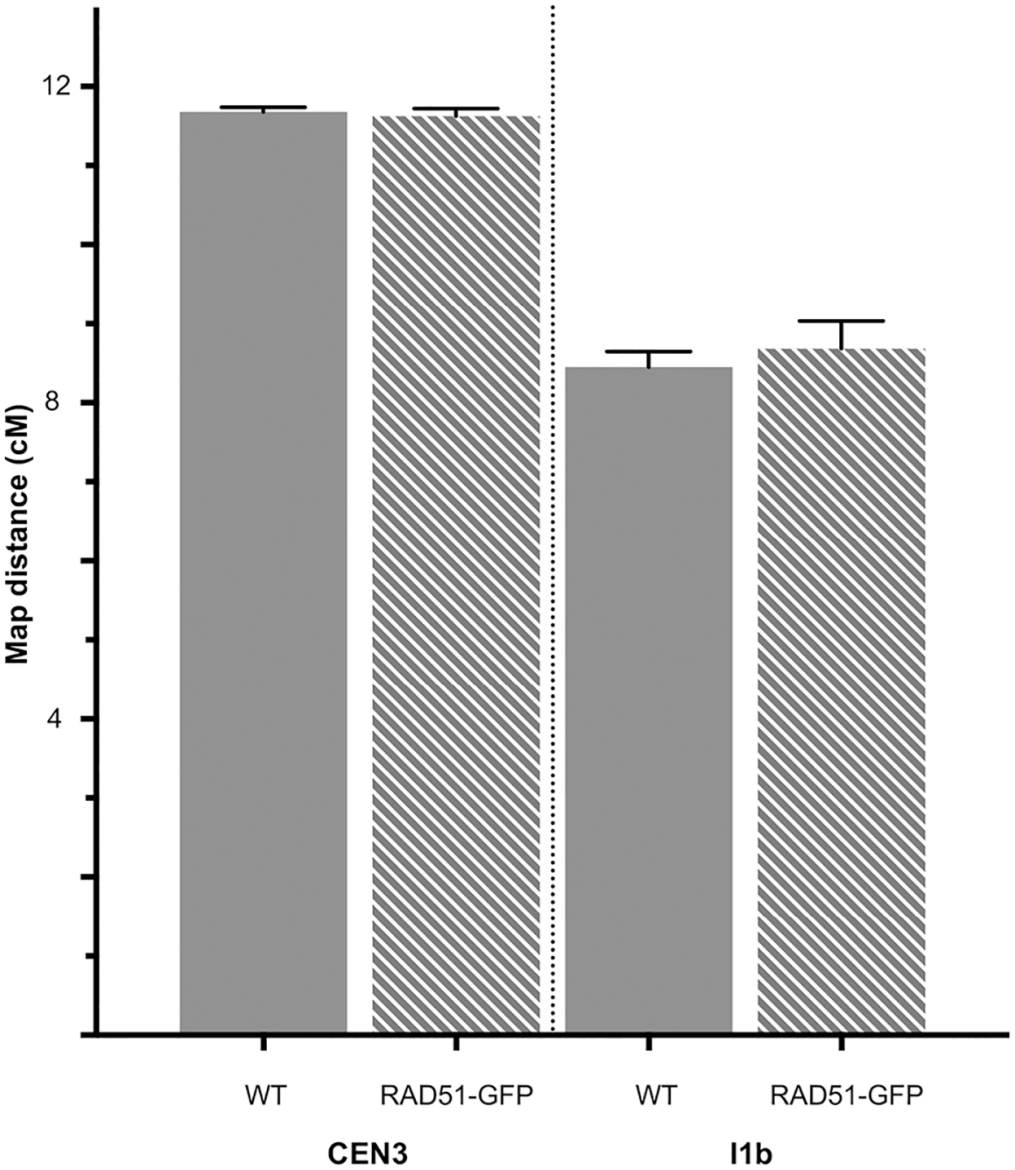
Genetic map distances of the I1b and CEN3 intervals in WT and *RAD51-GFP* meioses. Mean map lengths (cM) of the I1b and CEN3 genetic intervals in Wild type (filled bars) and *RAD51-GFP* plants (striped bars). Error bars are standard errors of the mean.

As expected and in agreement with our previous data on different chromosome-arm genetic intervals [31], absence of RAD51 strand exchange activity had no detectable effect on meiotic CO rate in the chromosome I1b interval (Fig 1, Table 2; WT: mean±sem = 8.45±0.20 cM; 6 plants, total pollen scored = 6261; RAD51-GFP: mean±sem = 8.68±0.35 cM; 6 plants, total pollen scored = 6923. unpaired 2-tailed t-test. P = 0.5751 t = 0.5795 df = 10). Neither was any significant effect of the absence of RAD51 strand-exchange activity observed in the centromere-spanning chromosome 3 interval, CEN3 (Fig 1, Table 1; WT: mean±sem = 11.68±0.06 cM; 5 plants, total pollen scored = 6304; RAD51-GFP: 11.62±0.10 cM; 5 plants, total pollen scored = 5142. unpaired 2-tailed t-test. P = 0.6103 t = 0.5303 df = 8).

These results concord with our previous measurements on 2 genetic intervals defined by INDEL markers on the arms of chromosomes I and III [31], showing no significant effect of the absence of functional RAD51 strand-exchange activity on meiotic CO rates in chromosome arms or across the centromere of Arabidopsis chromosome 3.

### Chiasmata counting

**Fig 2:**
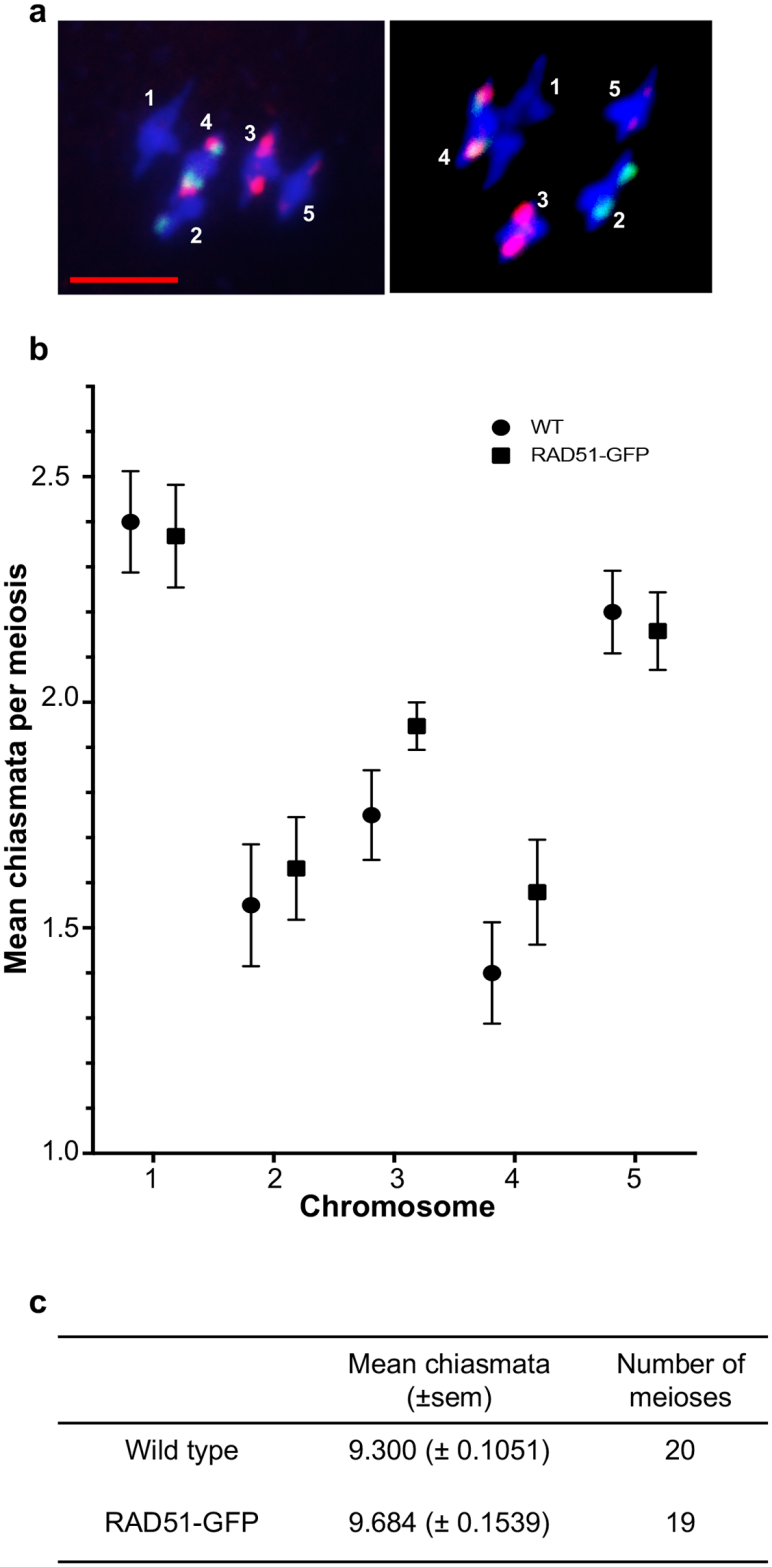
Chiasmata counts in wild type and RAD51-GFP meioses. DAPI-stained (blue) meiotic Metaphase I of wild type (a, left panel) and RAD51-GFP (a, right panel). Green (45S rDNA) and red (5S rDNA) FISH signals are used to identify each of the 5 chromosomes (numbered) and the shape of the bivalents permits counting chiasmata. Scale bar is 5μm. Mean numbers of chiasmata per chromosome (b) in wild type (blue) and RAD51-GFP (red) and per meiosis (c) (errors are s.e.m.).

Fluorescence *in situ* hybridisation (FISH) using probes for the 5S and 45S rDNA loci [55], permits identification of all 5 Arabidopsis chromosomes in meiotic metaphase I and the form of the bivalents can be used to infer mean CO numbers per chromosome and per meiosis (Fig 2a) [55].

Counting chiasmata showed means of 9.3 ± 0.11 (mean ± s.e.m.) and 9.68 ± 0.15 chiasmata per meiosis in Col-0 (wild type) and RAD51-GFP plants respectively (Fig 2c and Table 3). The absence of RAD51 strand exchange activity thus results in a mild increase in CO of borderline significance (unpaired 2-tailed t-test. P = 0.045, t = 2,08 df = 37). Taking the five chromosomes individually, numbers of chiasmata numbers per chromosome showed no significant differences between wild-type and RAD51-GFP plants (adjusted P values of 0.957, 0.957, 0.383, 0.725, 0.957 for chromosomes 1 to 5 respectively. Fig 2b, Table 3).

**Table 3:** Chiasmata counts.

	WT	RAD51-GFP	P	significant?
Chr 1	2.4±0.11	2.37±0.11	0.957	no
Chr 2	1.55±0.14	1.63±0.11	0.957	no
Chr 3	1.75±0.10	1.95±0.05	0.383	no
Chr 4	1.4±0.11	1.58±0.12	0.725	no
Chr 5	2.2±0.09	2.16±0.09	0.957	no
all	9.3 ± 0.11	9.7 ± 0.15	0.0445	yes*

Mean (±s.e.m.) numbers of chiasmata per chromosome and per meiosis in WT (N = 20) and RAD51-GFP (N = 19) plants. Adjusted P values (unpaired 2-tailed t-tests, Holm-Sidak method) show no significant differences for the chromosomes taken individually. A small difference of borderline significance is seen in the per-meiosis counts (*unpaired 2-tailed t-test. P = 0.045, t = 2,08 df = 37).

### Meiotic HEI10 foci

**Fig 3:**
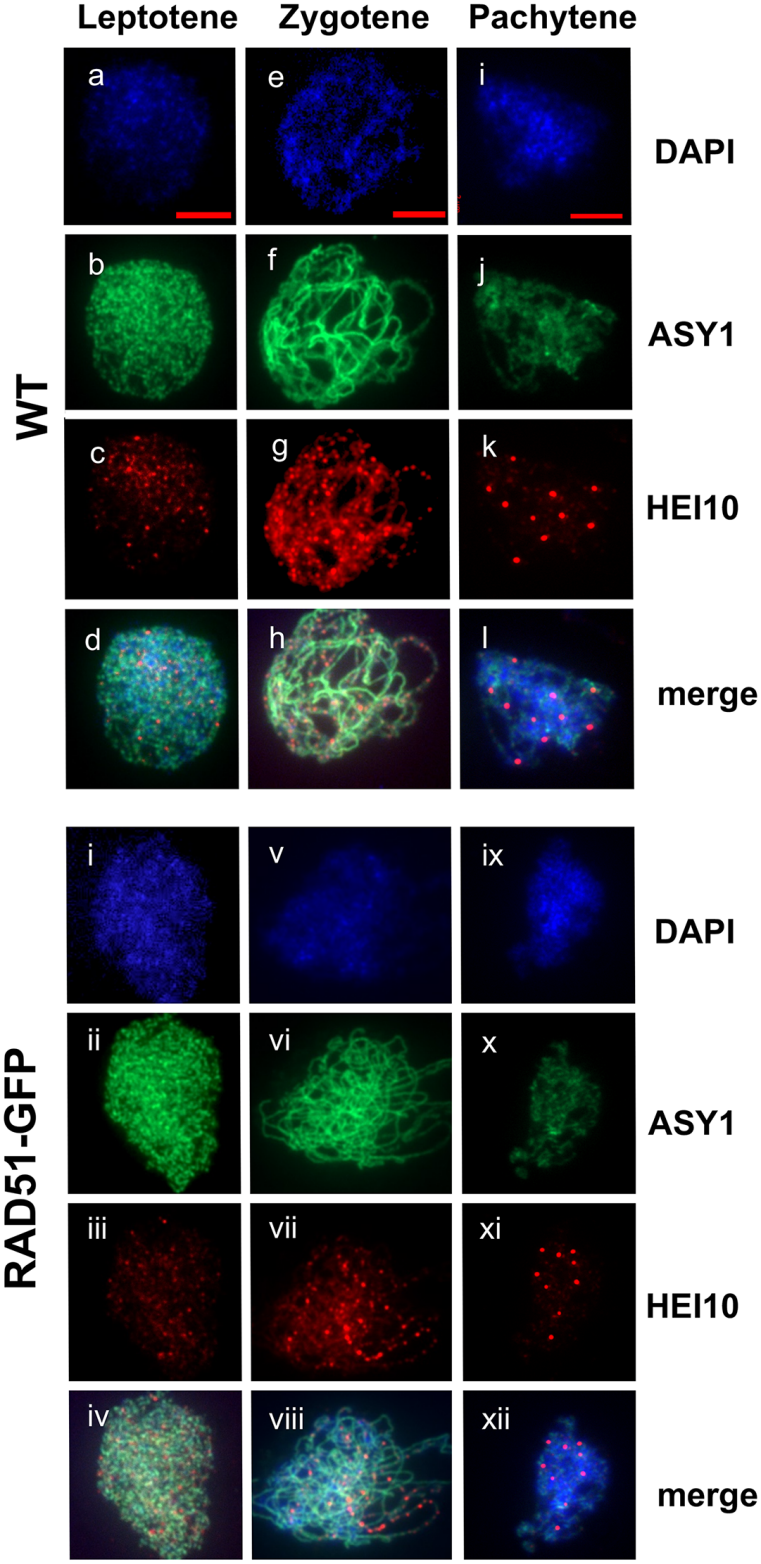
HEI10 foci in wild type and RAD51-GFP Pachytene. Immunolocalization of the ZMM protein HEI10 (red) and the meiotic protein ASY1 (green) in wild type Leptotene (a-d), Zygotene (e-h) and Pachytene (i-l) and RAD51-GFP Leptotene (i-iv), Zygotene (v-viii) and Pachytene (ix-xii) Pollen Mother Cell nuclei. Scale bar is 5μm.

Arabidopsis HEI10/ZYP3 is structurally and functionally related to yeast Zip3 and mammalian HEI10 and is required for the formation of Type I COs [56]. HEI10 immunolocalization (IF) foci can be used to quantify numbers of Type I COs. Fig 3 shows representative IF images of WT (a-d) and RAD51-GFP (e-h) Arabidopsis pollen mother cells (PMC) spreads with DAPI (blue), anti-ASY1 (green) and anti-HEI10 (red). As expected [56], the numbers of HEI10 foci visible on chromosome axes increase through leptotene into late zygotene in both wild type and RAD51-GFP and drop dramatically to give 7–11 foci/nucleus in late Pachytene. Mean (±s.e.m, number of meioses counted) numbers of HEI10 foci in Leptotene, Zygotene and Pachytene were 72.43 (±1.50, n = 7), 140.5 (±1.83, 10) and 9.5 (±0.183, n = 40) respectively for WT meioses. Leptotene, Zygotene and Pachytene values for RAD51-GFP meioses were 70.29 (±2.00, n = 7), 139.7 (±1.67, 10) and 9.73 (±0.168, n = 48) respectively. No significant differences were thus observed in numbers of HEI10 foci between WT and RAD51-GFP (2-tailed t-tests. Leptotene: P = 0.41, t = 0.859, df = 12; Zygotene: P = 0.750, t = 0.3234, df = 18; Pachytene: P = 0.382, t = 0.924, df = 86).

### Meiotic time-course

Previous reports have shown that perturbation of homologous recombination and synapsis causes delays in meiotic prophase I with, for example, the *zyp1* mutant causing an extension of prophase I by approximately 6 hours [57]. We thus tested for effects of the absence of RAD51 strand-exchange activity on the progression of the meiotic division using an EdU pulse-chase (see Methods). Briefly, a pulse of the thymidine analogue EdU is taken up through the transpiration stream and incorporated into DNA in replicating cells, including those in pre-meiotic S-phase. Anthers are collected and fixed across a time-course, and meiotic Pollen Mother Cell nuclei observed for the first occurrence of EdU labeled chromosomes at specific meiotic stages.

**Fig 4:**
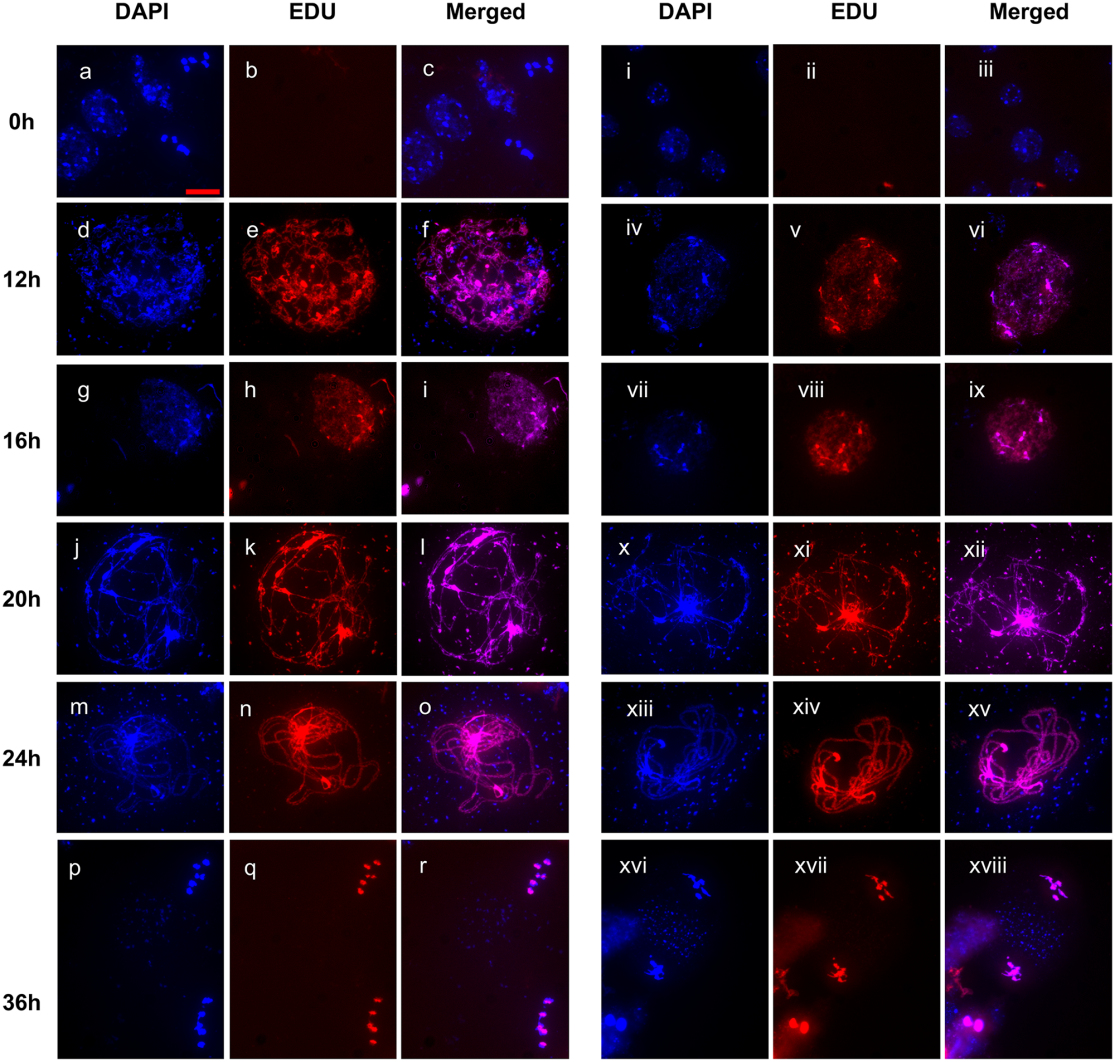
EdU pulse-chase meiotic time-course in wild type and RAD51-GFP plants. Wild type (a-r) and RAD51-GFP (i-xviii) pollen mother cells are in the pre-meiotic S/G2-phase 2 hours after the EdU pulse (+2 h), in leptotene at +12h, early zygotene at +16h, zygo-pachytene at +20h, pachytene at +24h and meiosis II at +36h. Scale bar is 10μm.

Meiocytes that incorporated EdU into their replicating DNA at the end of S-phase took approximately 6–8 hours to progress through G2 into early leptotene [58]. As seen in Fig 4, EdU signal was observed in leptotene nuclei 12 hours following the EdU pulse (Fig 4, panels d-f and iv-vi). EdU signal was detected in chromosomes of early zygotene meiocytes 16 hours after the pulse (Fig 4, panels g-i and vii-ix). At the 20h point, labelled chromosomes were observed in Zygotene/Pachytene (Fig 4, panel j-l and x-xii). At 24h Pachytene chromosomes were completely labelled with EdU (Fig 4, panel m-o and xiii-xiv), At 36h, EdU staining is visible only in meiosis II in both wild type and RAD51-GFP plants (Fig 4 panels p-r and xvi-xviii). EdU labelling thus followed the same kinetics in RAD51-GFP and WT plants, showing that the absence of RAD51 strand-exchange activity thus caused no detectable differences in the timing of meiotic stages in this analysis.

## Discussion

Notwithstanding the similar activities of the two proteins, *rad51* and *dmc1* mutants have very different meiotic phenotypes and the Arabidopsis *rad51* and *dmc1* mutants provide a very clear illustration of these differences. Accumulation of unrepaired meiotic DSB leads to Mek1-dependent meiotic arrest in the yeast *dmc1* mutant [3, 12, 59, 60]. The Arabidopsis *dmc1* mutant is however able to fully repair meiotic DSB created by the SPO11 complex, but has achiasmate meiosis and fertility is reduced to only a few percent of that of wild type plants. In striking contrast, the Arabidopsis *rad51* mutant is sterile due to chromosomal fragmentation in meiotic prophase I. In the absence of RAD51 protein, DMC1 alone is thus unable to repair meiotic DSB, while RAD51 (in the absence of DMC1) does repair meiotic DSB but without generating interhomologue CO and chiasmata [50, 51, 61, 62]. The dependence of DMC1 on the presence of RAD51 can also be seen in increased numbers of univalents and non-homologous chromosome associations caused by the Arabidopsis *rad51-2* knock-down allele [63] and the partial suppression of the *rad51* phenotype in the absence of ATR kinase [61]. The key to answering these puzzling differences came from the demonstration that inactivation of the secondary DNA binding site of RAD51 did not affect the fertility of *rad51-II3A* mutant yeast [30], nor RAD51-GFP in Arabidopsis [31]. The mutant *rad51-II3A* and RAD51-GFP proteins are unable to catalyse invasion of the template DNA duplex and are defective in mitotic DSB repair, but remain able to support the activity of DMC1 in meiosis [30, 31, 52].

DMC1 is thus capable of catalysing the repair of all meiotic DSB in the absence of RAD51 strand-exchange activity, but the question remains as to whether it does so in wild type meiosis or whether this result is specific to the *rad51*-mutant context. Given the excess of meiotic DSB over CO and the long-standing belief that the involvement of DMC1 in the repair of a given meiotic DSB was the key to it potentially resulting in a CO, both yeast and plant studies tested for effects on meiotic CO rates. The absence of detectable effects on CO patterns in yeast *rad51-II3A* [30] and Arabidopsis RAD51-GFP [31], suggested that this is the case. In this work we have taken advantage of the 25- to 30-fold excess of meiotic DSB over CO in Arabidopsis to extend our previous results on the possible effects of absence of RAD51 strand-exchange activity on meiotic CO patterns [31]. Compared to only 44% in budding yeast, more than 95% of meiotic DSB give rise to non-CO outcomes in WT Arabidopsis, making the plant a sensitive model to test for changes in their metabolism. Extending our previous results to more genetic intervals and to whole-chromosome and whole-genome measurements of chiasmata, we find no evidence for any significant effect in the absence of RAD51 strand-exchange activity on CO numbers or meiotic progression. This work thus extends and confirms the earlier yeast and Arabidopsis studies—arguing that DMC1 is the unique active meiotic strand-exchange protein in WT meiosis and thus appears to be responsible for intersister and inter-homologue CO, and very probably conversion.

## Materials and methods

### Plant material

All plants used in this study are of the Columbia ecotype of *Arabidopsis thaliana*. The *rad51-1* RAD51-GFP plant has been previously described [31]. The fluorescent pollen marked lines CEN3 and I1b [54] were kindly provided by Ian Henderson.

Seeds were sown in soil, stratified for two days at 4°C and grown in plant growth cabinets (SANYO MLR-351H) under standard conditions (16h day, 23°C, humidity 50–60%).

### Analysis of meiotic recombination rates

FTL marker lines [53, 54] were used to test for effects of the absence of RAD51 strand exchange activity on meiotic CO rates in peri-centromeric regions. The I1bc line carries three linked insertions on the right arm of chromosome 1 (FTL567, FTL1262, and FTL992). The CFP marker (FTL992) did not however yield repeatable results in our hands and so the I1b interval (FTL567:FTL1262 = 8.16 cM) was used in this work. The CEN3 line has two markers spanning the centromere of chromosome 3 (CEN3: FTL3332:FTL2536 = 10.43 cM—11.06 cM) [54, 64]). The I1b and CEN3 lines were crossed with Col-0 WT and *rad51/rad51* RAD51-GFP/RAD51-GFP homozygotes to generate F1 mapping lines heterozygous for the pollen markers in coupling, in which both DMC1 and RAD51 (WT), or only DMC1 (*rad51* RAD51-GFP) strand exchange activities are present during meiosis. Seeds of these plants were sown and the F2 plants genotyped to identify the homozygote F2 mapping lines for collection of pollen. The *rad51* KO allele and RAD51-GFP insertion were followed by PCR genotyping [31] and presence of the fluorescent markers was scored by visual examination of the pollen from flowers of the principal stems with a fluorescence microscope [53, 54].

### FISH

Meiotic chromosome spreads were prepared according to [55]. Briefly, whole inflorescences were fixed in ice-cold ethanol/glacial acetic acid (3:1) and stored at -20°C until further use. Immature flower buds of appropriate size were selected under a binocular microscope, rinsed twice at room temperature in distilled water for 5 min followed by two washes in 1X citrate buffer for 5 min. Flower buds were then incubated for 2 h on a slide in 100μl of enzyme mixture (0.3% w/v cellulase, 0.3% w/v pectolyase, 0.3% cytohelicase (Sigma)) in a moist chamber at 37°C. Buds were softened for 1 minute in 15μl 60% acetic acid on a microscope slide at 45°C, fixed with ice-cold ethanol/glacial acetic acid (3:1) and air dried. Finally, slides were mounted in Vectashield mounting medium with DAPI (Vector Labs. Burlingame, CA, USA) for microscopy.

### Immunocytology

Slide preparation for immunolocalization of proteins were carried out as described by [65]. Anti-ASY1 from Guinea-Pig (1:250 dilution) [66] and HEI10 from Rabbit (1:150 dilution) [56] were kindly provided by Chris. Franklin (Univ. Birmingham, U.K.) and Mathilde Grelon (INRA, Versailles, France).

### Microscopy

All observations were made with a motorised Zeiss AxioImager Z1 epifluorescence microscope (Carl Zeiss AG, Germany) using a PL Apochromat 100X/1.40 oil objective, AxioCam Mrm camera (Carl Zeiss AG, Germany) and appropriate Zeiss filter sets: 25HE (DAPI), 38HE (Alexa 488), 43HE (Alexa 596).

### Pulse chase experiment

Floral stems (approx. 8cm) of well-grown, 6 week-old *rad51/rad51 RAD51-GFP/RAD51-GFP* and WT plants [58, 67] were cut under running tap water and transferred in 10 mM EdU for 2h (Click-IT assay kit Invitrogen, California, USA). The floral tips were then rinsed under running water for 2–3 times and transferred to glass tubes containing tap water and incubated at 23°C, ~100–120μm/m^2^/s^-1^ light intensity). Samples were collected at 0h, 12h, 16h, 24h & 36h time points and fixed in ethanol: glacial acetic acid (3:1 ratio) and stored at 4°C. Meiotic chromosome spreads were prepared and stained and analysed as described [68, 69].

## Supporting information

S1 Fig Scoring fluorescent pollen. YFP (a), RFP (b), bright-field and merged (d) images of pollen from CEN3xCol-0 F1 plants carrying the fluorescent markers. Examples of the different combinations of fluorescence are arrowed. Scale bar is 5μm.
(PDF)
