# Supplementary material for: Analysis of the impact of the absence of RAD51 strand exchange activity in Arabidopsis meiosis
Source: PLoS One. 2017 Aug 10;12(8):e0183006. doi: 10.1371/journal.pone.0183006 (PMC5552350; doi:10.1371/journal.pone.0183006)
Supplement: S1 Fig — YFP (a), RFP (b), bright-field and merged (d) images of pollen from CEN3xCol-0 F1 plants carrying the fluorescent markers. Examples of the different combinations of fluorescence are arrowed. Scale bar is 5μm. (PDF) [file pone.0183006.s001.pdf]

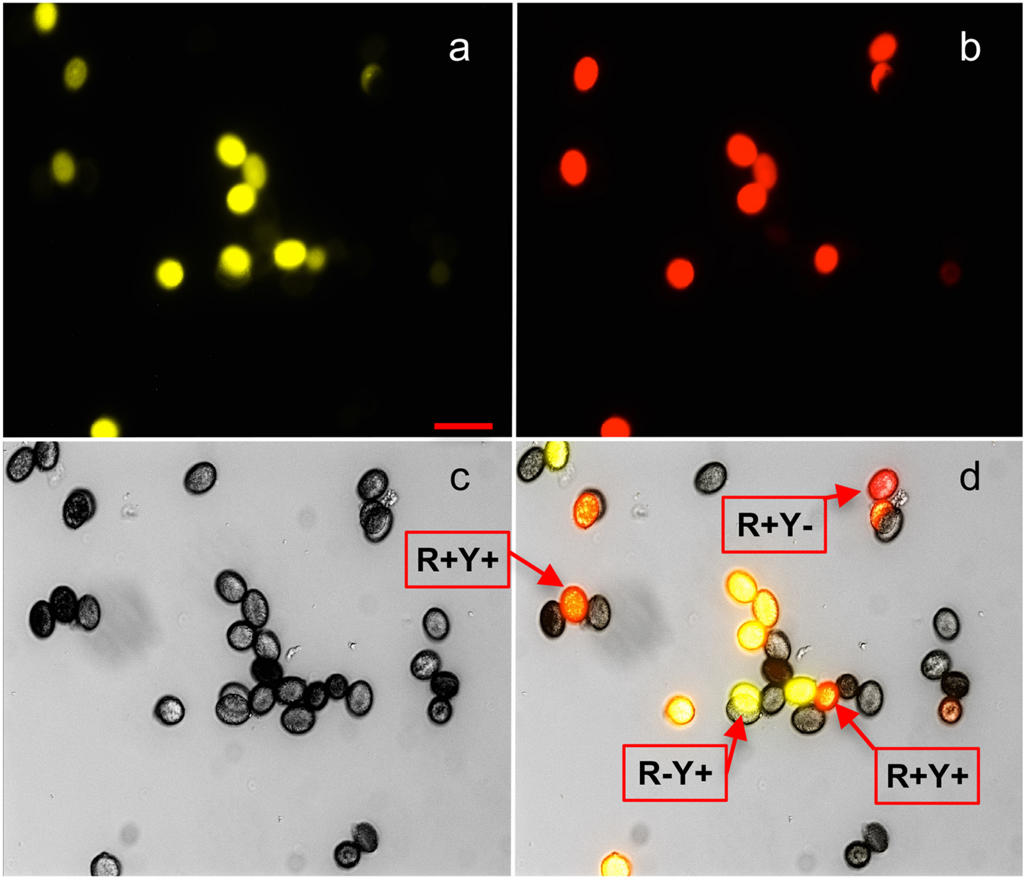

Supporting Figure 1. YFP (a), RFP (b), bright-field and merged (d) images of pollen from CEN3xCol0 F1 plants carrying the fluorescent markers. Examples of the different combinations of fluorescence are arrowed. Scale bar is 5µm.
